# Evaluation of the effectiveness of a novel mouth rinse containing hyaluronic acid and hydrogen peroxide on gingivitis: A randomized pilot controlled trial

**DOI:** 10.1002/cre2.498

**Published:** 2022-02-22

**Authors:** Elisa Boccalari, Santosh Kumar Tadakamadla, Chiara Occhipinti, Valentina Lanteri, Cinzia Maspero

**Affiliations:** ^1^ Department of Biomedical, Surgical and Dental Sciences University of Milan Milan Italy; ^2^ School of Medicine and Dentistry & Menzies Health Institute Queensland Griffith University Gold Coast Queensland Australia; ^3^ Fondazione IRCCS Ca' Granda Ospedale Maggiore Policlinico Milan Italy

**Keywords:** gingivitis, hyaluronic acid, hydrogen peroxide, mouthwashes

## Abstract

**Objective:**

To evaluate and compare the effectiveness of a mouth rinse with hydrogen peroxide (H_2_O_2_) and hyaluronic acid (HA) versus a placebo mouth rinse on gingivitis.

**Material and methods:**

This was a 21‐day, double blind, randomized, two‐arm parallel allocation placebo‐controlled trial of 50 study participants with a diagnosis of plaque‐induced gingivitis. Patients were randomly allocated to the test group or the placebo group and were assessed at three time points over the course of the study by the same operator, at baseline (T0), 7 days (T1), and 21 days follow‐up (T2). Primary outcomes of the trial were improvement in gingivitis and plaque accumulation.

**Results:**

In both groups there was a decrease in gingival and plaque indices but the combination of the two actives (H_2_O_2_/HA) proved to be more effective against gingivitis (*p* = 0.001). Regarding plaque index, the differences between the test product and placebo were not statistically significant (*p* = 0.084). Besides, the new mouthwash was considered palatable, no adverse events were registered over the 21‐day period.

**Conclusions:**

The H_2_O_2_ + HA product was more effective in reducing gingivitis when compared to a placebo while no differences were observed for plaque accumulation.

## 1 INTRODUCTION

Gingivitis is an inflammatory condition of the gum that might clinically manifest with bleeding (spontaneous or after periodontal probing), gingival hypertrophy, erythema or oedema of the affected tissues, but does not involve loss of supporting tissue of the tooth (Chapple et al., 2018). Its most common form is caused by bacteria in association with the presence of plaque although it can also be induced by viruses, fungi, trauma, allergic reactions to dental material, immune conditions. Other risk factors include poor dental hygiene, smoking as well as metabolic, nutritional, and genetic factors (Lindhe & Lang, 2015).

Gingivitis is a reversible condition but, if not properly treated and in susceptible subjects, it can develop into periodontitis. Prevention of gingivitis is certainly to be considered as the main form of prevention of periodontitis. It can be achieved through the control of its causative agent, bacterial plaque (Chapple et al., 2015). This can be accomplished both at home and with dental chair treatments (with dentists and dental hygienists), through control of the supragingival plaque.

Regarding home treatment, motivation and instruction of patients are fundamental in order to learn how to correctly use toothbrush and interdental hygiene instruments. Manual and power brushing significantly reduces plaque levels and gingival inflammation. Moreover, antiplaque agents in the form of mouthwashes are useful in association with conventional tooth brushing (Chapple et al., 2015).

In fact, the difficulty of achieving and maintaining high levels of home oral hygiene with tooth brushing and interdental cleaning has prompted the researchers and industry to identify ideal pharmacological agents to control plaque formation and gingival inflammation (Tadakamadla et al., 2019).

Pharmacological agents contained in some mouthwashes are antimicrobials that act on the bacterial plaque to prevent its formation. Some agents that have been proven to be effective in plaque control are chlorhexidine, essential oils, fluoride, and phenols when used as mouth rinses (Azaripour et al., 2016; Lynch et al., 2018; Marchetti et al., 2017).

The novel product to be tested in this study is a solution enriched with hydrogen peroxide (H_2_O_2_) and hyaluronic acid (HA). HA is a glycosaminoglycan and one of the main components of the extracellular matrix, it can be found in different tissues such as skin, cartilage, synovial fluid, tendons, eyes. It has an important role in tissue repair processes and it modulates cell–cell and cell–matrix interactions, tissue hydration and angiogenesis. It also promotes the development of granulation tissue and stimulates cell proliferation, migration and differentiation (Chen & Abatangelo, 1999; Dechert et al., 2006).

HA has been proved to be effective in inhibiting plaque growth in an in vivo study, similarly to chlorhexidine (Rodrigues et al., 2010), and in reducing pain, thanks to its hydrating and film forming abilities (Lopez‐Jornet et al., 2010; Tartaglia et al., 2017). It not only helps protecting the oral mucosa but also allows retention of other actives at the site of action to prolong their effect. In addition, HA in combination with cetylpyridinium chloride resulted to be as effective as chlorhexidine in preventing plaque (Tadakamadla et al., 2019). It also seems to be effective in reducing the inflammatory response if used as an adjunct to nonsurgical and surgical periodontal therapies demonstrated through clinical parameters such as bleeding on probing (BOP) and probing depth (PD) (Bertl et al., 2015; Eliezer et al., 2019).

The other component of the solution is H_2_O_2_, which has a strong oxidation capacity and pro‐inflammatory activity able to disinfect wound tissues (in solution of 0.5%–3%). Recent studies demonstrate that it also plays multiple functions in wound healing. Although high levels of H_2_O_2_ could cause oxidative damage leading to delayed healing, low concentrations of H_2_O_2_ are thought to promote healing (Loo et al., 2012). It also upregulates the expression of inflammation related genes and the synthesis of proinflammatory cytokines, including TNF‐α, IL‐1β, IL‐5 (Cui et al., 2016).

Moreover, it plays a role in the inflammatory phase too, inducing chemotaxis and adherence of neutrophils and macrophages (Sen & Roy, 2008). These have bactericidal activity and eliminate any microorganisms through the formation of proteases and elastases. Moreover, at low concentrations (250 μM) H_2_O_2_ promotes re‐epithelialization through keratinocyte migration in the healing site (Loo & Halliwell, 2012).

In dentistry H_2_O_2_ was traditionally usually used as a bleaching agent but it has also been revised its use as a mouthwash, alone or combined with chlorhexidine (CHX), in the prevention of plaque and reduction of inflammation but the results of its efficacy from the studies are mixed (Hossainian et al., 2011; Kamolnarumeth et al., 2021).

This study aims to evaluate the efficacy of the novel mouth rinse containing H_2_O_2_ and HA on gingivitis and plaque accumulation in comparison to a placebo product. In addition, the safety and acceptability of the novel mouth rinse will be evaluated.

## 2 MATERIAL AND METHODS

### 2.1 Study design and population

This was a two‐arm double‐blinded, randomized, placebo controlled, parallel‐group 21‐day study with random allocation of patients to the two groups: the first one received a H_2_O_2_/HA mouth rinse (BMG Pharma, Milan, Italy) and the second one a hydro based placebo. The protocol of this clinical trial was registered on ClincalTrails.gov and is accessible at https://clinicaltrials.gov/ct2/show/NCT04438421. Ethics approval was granted by the institutional ethics committee of the IRCCS Ca' Granda, Ospedale Maggiore Policlinico of Milan, Italy (nr. 21/19).

As this is the first study that is testing this novel combination of the mouth rinse, we have used the effect size from a previous study that tested a combination of HA and cetylpyridinium chloride (CPC) (Tadakamadla et al., 2019). A sample size of 48 (24 in each group) was calculated to be adequate with a type I error of 5% and power of 80% assuming an effect size of 0.84 for plaque.

Fifty participants were enrolled, 23 males and 27 females with gingivitis caused by accumulation of plaque. Study participants were consecutively selected from the outpatients attending the dental clinic hygiene program at the IRCCS “Ca” Granda Ospedale Maggiore Policlinico of Milan—UOC Maxillo Facial Surgery and Dentistry and assessed for eligibility.

Study participants older than 18 years old with plaque induced gingivitis, willing to provide informed consent, were considered for inclusion.

Plaque induced gingivitis was diagnosed if patients had a probing pocket depth (the distance from the gingival margin to the bottom of the periodontal pocket) of ≤3 mm and a bleeding on probing at ≥10% of the sites, on intact periodontium (Chapple et al., 2018).

Exclusion criteria included: patients suffering from systemic conditions such as diabetes mellitus, hypertension, hepatitis, HIV, acute and/or chronic infectious pathologies as well as those using topical or systemic drugs, smokers and patients unable to follow hygiene instructions during the intervention phase. There were no patients with fixed orthodontic appliances nor active carious lesions, overhanging fillings or crowns as all these patients were attending the dental hygiene clinic for routine scale and polish.

**Figure 1:**
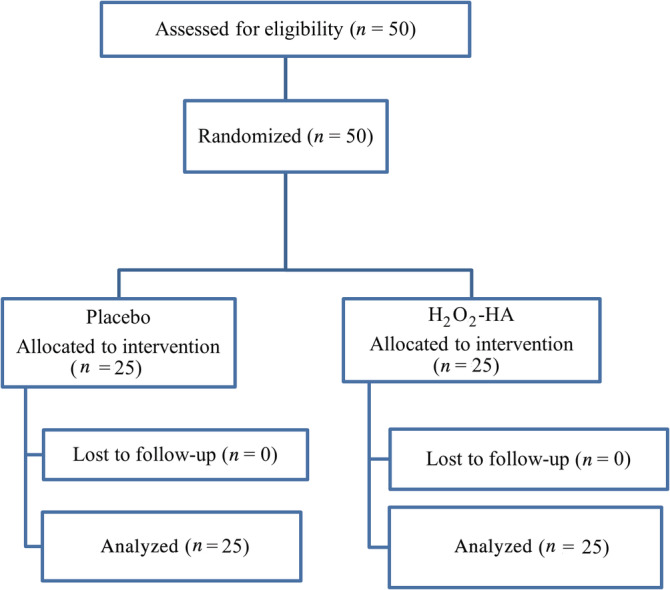
Flow chart depicting the participant recruitment

Patients received an information sheet describing the protocol and those interested provided a written consent. All eligible participants were subsequently equally randomized into the test or the control group (Figure 1).

### 2.2 Randomization and allocation concealment

A sequence of random numbers was created, by an external operator, using SPSS and each participant was randomly given a number and a corresponding kit containing the products. The products were packaged in such a way that they were not recognizable either by the operator or by the patient. Each package was, in turn, assigned a number that referred to the type of the product. The association between the number and the type of the product was collected by another operator, not related to the data collection. The data were collected in anonymised form and entered into a database in order to carry out the statistical analysis.

### 2.3 Clinical evaluation

The enrolled participants were examined at baseline (T0), 7 days (T1), and 21 days follow‐up (T2) by a single experienced dental clinician. Prior to the start of data collection, the examiner participated in a training session. The procedures of dental hygiene by means of ultrasonic device, at baseline, were performed by an expert dental hygienist. Study participants were then given the test or placebo product to be used twice a day, they were instructed to rinse for 30 s with 10 ml after normal oral hygiene procedures and were also advised not to drink or eat for 60 min after rinsing. The test product was a mouth rinse containing a combination of hydrogen peroxide (1,80%) and sodium hyaluronate (0,10%) with the remaining part being water (97,3%) and inert additives (BMG Pharma, Milan, Italy) while the placebo was a hydro‐based mouthwash (98.55% water and the remaining part comprising few inert additives). Patients were instructed to perform proper their regular oral hygiene regimen at home including interdental cleaning. Patients with allergic reactions or hypersensitivity due to the use of the products were advised to discontinue it and seek medical advice in order to assess the symptomatology and to undergo alternative therapies, if needed.

At baseline, 7‐ and 21‐day appointments, the outcomes of the study were assessed through two different indices.

A modified Silness and Loe plaque index (PI) was used to evaluate the level of plaque formation on six surfaces of each tooth (distobuccal, mesiobuccal, midbuccal, distolingual, mesiolingual, and midlingual). A score of 0 was given when the tooth surface was free of any supragingival plaque, while a score of 1 was given on presence of a film of plaque on the free gingival margin only visible after application of a disclosing solution or when using a probe. A score of 2 indicated a moderate presence of plaque on the tooth and gingival margin, visible with the naked eye. A score of 3 showed abundant presence of plaque extending 1–2 mm from the gingival margin (Silness & Loe, 1964).

Gingival inflammation was assessed using a modified version of Silness and Loe gingival index (GI), a method of numerically recording the extent of gingival bleeding on probing. Six surfaces per tooth were considered (distobuccal, mesiobuccal, midbuccal, distolingual, mesiolingual, and midlingual).

Gingival index was scored as 0 when the gingiva appeared normal on probing. Score 1 denoted mild inflammation with redness and oedema but absence of bleeding on probing; Score 2 indicated moderate inflammation with redness and oedema associated with bleeding on probing while Score 3 represented severe inflammation, redness and oedema, ulceration, and spontaneous bleeding (Löe, 1967).

In addition to objective clinical examination, patients were asked to report their perception of odor and taste of the product, through a scale ranging from 1 to 10. They were assessed at the beginning (T0), at 7 (T1), and 21 (T2) days follow‐up, with 1 being the worst odor and taste and 10 being the most pleasant. Twenty eight teeth per patient were considered.

Overall scores of plaque and gingivitis indices in each individual were calculated by dividing the sum of the scores of each tooth by the product of the total number of the analyzed teeth with the maximum score which can be reached by each tooth (namely 18). The result of this equation was multiplied by 100 and presented as a percentage.

### 2.4 Statistical analysis

Statistical analysis was carried out by an operator who did not know the type of treatment that the patients underwent.

Statistical analysis was performed through Stata 16.1 software. Unpaired *t*‐test was used to compare age, PI and GI scores at baseline between the two groups and Mann–Whitney test was used to compare odor and taste. Mixed regression model with robust errors was used to separately evaluate the change of GI and PI considering time, group and their interaction as fixed factor and patients as random factor. Estimated mean and SE were reported. Bonferroni correction was applied to comparison of T1 and T2 versus T0. Median and Interquartile range (Q1–Q3) of odor and taste were reported. Friedman test was used to evaluate change in odor and taste scores across the three time points. Statistical significance level was set at 0.05.

## 3 RESULTS

Fifty patients participated in the study, 23 males and 27 females, and they all completed follow up examination. Demographic characteristics of the participants are presented in Table 1.

**Table 1:** Demographics of participants

	H_2_O_2_/HA mouth rinse	Placebo
Number of study participants	25	25
Males (*N*, %)	12, 48.0%	11, 44.0%
Age in years (mean, SD)	35, 12.5	40, 13.4

## 4 PRIMARY OUTCOMES

**Figure 2:**
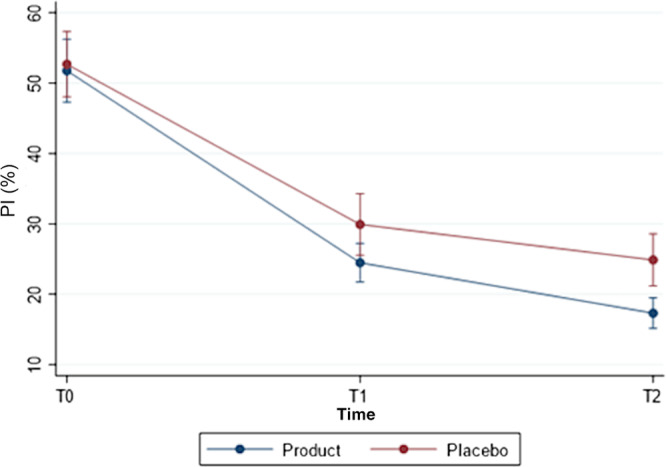
Plaque index score in relation to time‐point in the two groups (mean values, SD)

**Figure 3:**
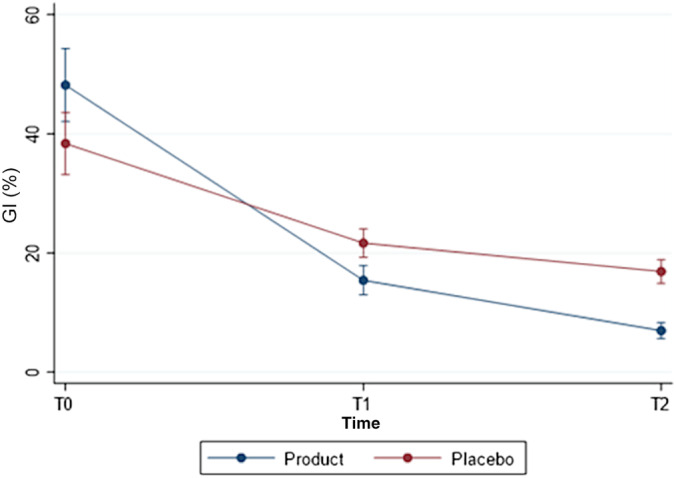
Gingival index score at baseline, seven and 21‐day time‐point in the two groups (mean values, SD)

Values of PI and GI significantly decreased in both groups (*p* < 0.001) (Figures 2 and 3). At baseline there were no statistical differences between the two groups regarding PI (*p* value = 0.783) with values ranging from 35 to 89 in the placebo group and from 36 to 78 in the H_2_O_2_/HA group. On the contrary, patients in the placebo group showed a significant lower GI score than those in the H_2_O_2_/HA mouth rinse group (*p* = 0.022, mean value of 38.4 in the placebo group versus 48.2 in the tested one, Table 2) with values ranging from 19 to 71 in the placebo group and from 25 to 85 in the test group. Regarding changes in PI from baseline to T2, no differences were observed between the groups (*p* = 0.084) with values at T2 in the H_2_O_2_/HA group ranging from 9 to 29 and from 8 to 39 in the placebo one. On the contrary, reduction in GI scores from baseline to T1 and T2 were significantly higher in the test group (H_2_O_2_/HA) than in the placebo (Table 3, *p* = 0.001). In the test group at T2 values were between 0 and 14 and ranged from 4 to 27 in the placebo one.

**Table 2:** Mean values at T0 of the examined variables

	H_2_O_2_/HA		Placebo		*p* Value
	Mean	SD	mean	SD	
PI	51.8	11.5	52.7	12	0.783
GI	48.2	15.8	38.4	13.4	0.022

**Table 3:** Estimated mean change from baseline

	H_2_O_2_/HA mouth rinse		Placebo		*p* ^a^
	Mean	SE	Mean	SE	
PI					
T1 vs. T0	−27.28	2.39	−22.76	2.33	0.352
T2 vs. T0	−34.44	2.29	−27.8	2.34	0.084
GI					
T1 vs. T0	−32.76	3.41	−16.72	2.21	0.001
T2 vs. T0	−41.2	3.21	−21.48	2.73	0.001

*Note*: Means, SE, and *p* values are estimated from mixed model with robust errors considering interaction from group and time as fixed factor and subjects as random factor.

^a^ Bonferroni correction, *p* value is related to the comparison of the mean change between the two groups.

## 5 SECONDARY OUTCOMES

No side effects were registered among the two groups during the 21 days. Although data about 10 patients in the placebo group are missing regarding odor and taste, we decided to include those study participants in our analysis since it was a secondary outcome.

Taste and odor scores significantly increased over time in the test group compared to the placebo group and participants using the H_2_O_2_/HA mouth rinse reported more pleasant odor and taste than the control group patients (*p* < 0.001, Table 4). Regarding taste, in the H_2_O_2_/HA group, the median was 8 at baseline, improving to 9 at T2, with a significant change over time (*p* < 0.001), while in the placebo group the median value remained unchanged but was significantly different from baseline (*p* = 0.021). Similarly, odor scores significantly changed in the test group (*p* = 0.034), while no changes were noted in the placebo group (*p* = 0.097).

**Table 4:** Taste and odor results in the two groups

	H_2_O_2_/HA mouth rinse		Placebo		*p* Value
	Median	Q1–Q3	Median	Q1–Q3	
Taste					
T0	8	7–9	5	2–6	<0.001^a^
T1	8	7–9	5	2–6	
T2	9	7–9	5	3–6	
	<0.001^b^		0.021^b^		
Odor					
T0	8	7.5–9	4	2–5	<0.001^a^
T1	8	7.5–9	5	2–6	
T2	9	7.5–9	5	2–6	
	0.034^b^		0.097^b^		

^a^ Mann–Whitney test.

^b^ Friedman test.

## 6 DISCUSSION

The aim of our study was to evaluate a new formulation with HA and H_2_O_2_ in the reduction of plaque accumulation and gingival inflammation in patients with plaque induced gingivitis. Both these actives have been independently studied, in vitro and in vivo, and their properties are well known. For instance, HA, according to Rodrigues et al. (Rodrigues et al., 2010), showed an antibacterial effect, reducing the growth of two of the most common periodontopathogens, *Aggregatibacter actinomycetemcomitans* and *Prevotella intermedia*, similarly to chlorhexidine. On the other hand, a recent literature review confirmed H_2_O_2_ anti‐bacterial, anti‐inflammatory and anti‐plaque effects which have been debated for several years (Muniz et al., 2020). Chlorhexidine remains to be considered as gold standard in terms of control of gingivitis and plaque in adjunct to mechanical tooth brushing and other hygiene procedures. However, it has several potential local side effects, such as staining, which is the most common, supragingival calculus accumulation, oral lesions and an altered taste perception (Tartaglia et al., 2019). Hence, the need for an alternative mouthwash, with comparable results but without the aforementioned side effects.

Since this is a new product, the first one which combined HA and H_2_O_2_, a direct comparison with other studies is not possible. The results of our study showed that there was a statistically significant difference between the placebo and the test groups at T2 in relation to GI. In fact, patients using the H_2_O_2_/HA mouth rinse had lower levels of gingivitis when compared to the placebo group. These data are in accordance with one of the few previous report analyzing a formulation with HA (Abdulkareem et al., 2020). Abdulkareem et al. (2020), evaluating a different formulation with 0.025% HA in comparison with a 0.12% chlorhexidine mouth rinse and a placebo found that HA caused a significant decrease in gingival inflammation, compared to baseline and the placebo. Clark et al. tested a mouthwash with H_2_O_2_ and Povidone‐iodine (PVP‐I) in a 6‐month randomized controlled trial and it proved to be effective in reducing gingival inflammation when compared to a placebo control (Clark et al., 1989).

Regarding PI, our data showed that the differences among the two groups were not statistically significant. This lack of significance in PI values is in contrast with Abdulkareem et al. (2020), who found a significant decrease in plaque among 0.025% HA users after 7 days, though CHX group demonstrated its superiority over HA. Tadakamadla et al. (2019), while studying a formulation with HA and CPC reported significantly higher mean values of plaque in the placebo group compared to the HA + CPC and CHX groups, in a 21 days clinical trial.

Detailed instructions for routine oral hygiene procedures were provided by the operator at T0 which would have led to an adequate mechanical action of tooth brushing. This could be demonstrated through low scores of plaque and gingivitis at follow‐up even in the placebo group, thus indicating not optimal levels of oral hygiene of the patients. This may have influenced the results, masking the effects of the test mouth rinse and thus the lack of significance in PI levels in our sample.

No side effects were reported by the patients during the trial period; unlike the most common mouthwashes, which contain chlorhexidine, tooth staining is not a side effect of this new formulation (Tartaglia et al., 2019). There were no reports of soft tissue lesions either, thanks to the low concentrations of H_2_O_2_ used, in accordance with previous literature (Walsh, 2000).

Secondary outcomes of this study were the perception of both taste and odor of the product over time. Among participants using H_2_O_2_/HA, odor slightly improved over time and it was considered pleasant despite the pungent smell which usually characterized H_2_O_2_. On the other hand, the odor scores for placebo formulation did not significantly change. Regarding taste, the test product seemed palatable and patients' satisfaction slightly increased between T0 and T2, and as they became familiar with it participants using H_2_O_2_/HA mouth rinse reported more pleasant odor and taste than the control group patients at all time‐points.

Limitation of our study included the unequal distribution of GI levels at T0 among the two groups, with the test group having higher values of GI at baseline compared to the placebo one. Another limitation is the lack of a positive control group, i.e. patients using chlorhexidine, thus limiting the validation of our product. Moreover, the long effectiveness of our test product could not be evaluated since the study lasted for 21 days.

In conclusion, despite the limitations of our study the novel mouth rinse with HA and H_2_O_2_ proved to be palatable and with a pleasant smell. Compared to the placebo product, use of the test product caused a reduction in gingival inflammation, while no differences were observed regarding plaque control. It can be considered an important aid, along with mechanical toothbrushing, in maintaining healthy gums, thus allowing the resolution of gingivitis and avoiding its subsequent problems. The promising results of this first study warrant the need to conduct a RCT to compare the efficacy of this new formulation with a chlorhexidine mouthwash, which is still considered to be the gold standard to prevent gingival inflammation and control plaque.

## CONFLICT OF INTEREST

The authors declare no conflict of interest.

## AUTHOR CONTRIBUTIONS

*Conceptualization*: Cinzia Maspero; *Supervision*: Santosh Kumar Tadakamadla; *Data collection*: Chiara Occhipinti; *Writing—original draft*: Elisa Boccalari; *Writing—review and editing*: Elisa Boccalari, Santosh Kumar Tadakamadla, Valentina Lanteri, and Cinzia Maspero.

## Data Availability

The data that support the findings of this study are available from the corresponding author upon reasonable request.
